# Hospital Architecture and Construction

**Published:** 1912-03-30

**Authors:** 


### HOSPITAL ARCHITECTURE AND CONSTRUCTION.

[Communications on this subject should be marked "Architecture" in the left-hand top corner of the envelope.]

### New Principles in the Design of Children's Hospitals.

One of the greatest difficulties which those in administrative or medical charge of hospitals for infants or children have to face, is that of infection and its prevention. In fever hospitals, or hospitals whose main object is the reception of cases of infective disease of any kind, the difficulty is, of course, great; but, at any rate, everyone is on the alert to prevent it, and at least the children are placed in wards where all have the same disorder, so that they infect neither each other nor those elsewhere suffering from different diseases.

### Infection and General Hospitals.

In general non-infectious hospitals the difficulties are enormously greater. A child is admitted for some illness thought not to be infective, and infects a whole ward, through a perhaps quite excusable mistake in diagnosis. More commonly still, a child is admitted, often on account of some deformity or other surgical condition, whilst in the incubation stage of some infection, and thus it defies detection. When the invasion period begins and the disease declares itself, the mischief is already done; and the infection spreads far and wide among the susceptible population to which it has obtained access.

These events are by no means uncommon; and the eradication of infection, when once it has obtained a firm grip on a hospital or ward, may be only possible by shutting up the whole area of the outbreak, disinfecting thoroughly, and reopening only after a considerable interval of time. New means of preventing or arresting such outbreaks of disease have been devised in late years; and the well-known New York physician, Dr. Henry Koplik, who discovered the valuable sign of commencing measles which is now known by his name, has lately published two papers giving his most recent observations and conclusions on the subject.*

To begin with, he emphasises the need for various precautions which have long been relied upon, though they are not always carried out as thoroughly as is advisable. The system of individual utensils for each infant or child hardly needs any emphasis; and amongst utensils all such articles as towels, flannels, bedding, and so forth are, of course, to be included. Wards for infants or children should be quite small.

It is, however, in methods of effective isolation and segregation that Dr. Koplik's views will chiefly interest hospital architects, managers, and medical officers. The so-called "box" system now so popular in France he does not altogether favour, on the ground that insufficient ventilation may result. The box system is, of course, as yet in an experimental stage. So is, no doubt, the system of glass partitions which has been adopted lately in two new hospitals in Germany. But Dr. Koplik thinks highly of it; and a short description of the Weiensee Hospital, near Berlin, may be instructive as showing what German architects are doing.

This hospital is a modification of the well-known Sauglingsheim at Munich. The blocks are so planned that each pavilion lies north and south; the patients' rooms face the south, the auxiliary offices face the north. Outside the wards is a broad covered veranda, which can be fully protected against the sun by well-arranged awnings.

The wards are arranged in pairs, each containing five beds, with service-rooms common to each pair. All the partitions are of glass to about four feet from the floor, so that standing in one ward a person can see what is going on throughout the pavilion. It may be added that even the linen-closet is constructed of glass, so that it can be seen whether it requires replenishing without entering the ward. The floors are covered with a green linoleum fixed down with a wax cement. The infants enter reception-wards, whence, after a sojourn for observation, they pass into permanent wards.

The great advantage of this system is that the advantages of the small ward are secured without that relatively enormous increase of the nursing staff which is necessary if the walls of the ward are opaque.

### The Glass-room Plan in Germany.

It is interesting to learn that the new children's pavilions at the Kaiser and Kaiserin Friedrich Hospital in Berlin have been constructed on the same principles. Here the rooms hold from two to six children each. Standing at one end of the corridor the whole series of rooms, divided by glass partitions, are visible, and the beds with them.

In Vienna, among the new clinics for children, some of the pavilions are constructed on the glass-room plan. But Dr. Koplik's description certainly bears out the contention that the ventilation of this type must be difficult and defective, and it is therefore to be hoped that the German models will be followed for preference. His testimony to the excellent state of the air-supply both at the Weiensee and the Kaiser and Kaiserin Friedrich is therefore valuable as showing hospital architects that the system is a practicable one; and it will be of great interest to see whether the glass-room ward succeeds in gaining a foothold in this country.

* *Archives of Pediatrics*, September 1911 and January 1912.

